# Prevalence and Clinical Attributes of Congenital Microcephaly — New York, 2013–2015

**DOI:** 10.15585/mmwr.mm6605a1

**Published:** 2017-02-10

**Authors:** Krishika A. Graham, Deborah J. Fox, Achala Talati, Cristian Pantea, Laura Brady, Sondra L. Carter, Eric Friedenberg, Neil M. Vora, Marilyn L. Browne, Christopher T. Lee

**Affiliations:** ^1^Public Health/Preventive Medicine Residency Program, Division of Epidemiology, New York City Department of Health and Mental Hygiene; ^2^Congenital Malformations Registry, New York State Department of Health; ^3^Division of Disease Control, New York City Department of Health and Mental Hygiene; ^4^Office of Public Health Preparedness and Response, CDC; ^5^Epidemic Intelligence Service, CDC.

Congenital Zika virus infection can cause microcephaly and other severe fetal neurological anomalies (*1*). To inform microcephaly surveillance efforts and assess ascertainment sources, the New York State Department of Health and the New York City Department of Health and Mental Hygiene sought to determine the prevalence of microcephaly in New York during 2013–2015, before known importation of Zika virus infections. Suspected newborn microcephaly diagnoses were identified from 1) reports submitted by birth hospitals in response to a request and 2) queries of a hospital administrative discharge database for newborn microcephaly diagnoses. Anthropometric measurements, maternal demographics, and pregnancy characteristics were abstracted from newborn records from both sources. Diagnoses were classified using microcephaly case definitions developed by CDC and the National Birth Defects Prevention Network (NBDPN) (*2*). During 2013–2015, 284 newborns in New York met the case definition for severe congenital microcephaly (prevalence = 4.2 per 10,000 live births). Most newborns with severe congenital microcephaly were identified by both sources; 263 (93%) were identified through hospital requests and 256 (90%) were identified through administrative discharge data. The proportions of newborns with severe congenital microcephaly who were black (30%) or Hispanic (31%) were higher than the observed proportions of black (15%) or Hispanic (23%) infants among New York live births. Fifty-eight percent of newborns with severe congenital microcephaly were born to mothers with pregnancy complications or who had in utero or perinatal infections or teratogenic exposures, genetic disorders, or family histories of birth defects.

Since early 2015, Zika virus has spread widely throughout the World Health Organization’s Region of the Americas (*3*). Zika virus infection during pregnancy can cause severe birth defects, including microcephaly, with the highest risk for adverse pregnancy outcomes associated with infection during the first trimester (*1*,*4*). Most Zika virus infections have been imported into the continental United States, with almost all local transmission reported from Florida (*5*). New York has recorded the largest number of travel-associated Zika virus disease cases^†^ in the continental United States; the majority of these have occurred in New York City. As of December 13, 2016, among 34 infants born in the continental United States with Zika-associated birth defects, five (15%) were born in New York City (*5*,*6*).

During 2009–2013, population-based birth defects surveillance programs estimated the median prevalence of microcephaly in the United States to be approximately 7 per 10,000 live births; however, case-finding methods and clinical definitions of microcephaly varied among states (*7*). Until recently, a microcephaly case definition had not been standardized across jurisdictions. In the wake of rapidly spreading Zika virus infection and its impact on birth outcomes, NBDPN, in conjunction with CDC, developed case definitions for congenital microcephaly for use by state birth defects surveillance programs (*2*).

Surveillance of birth defects in New York is conducted by the New York Congenital Malformations Registry (the Registry), a surveillance system that receives reports from hospitals on major birth defects in infants and children diagnosed before the age of 2 years. The Registry requires a narrative description of birth defects along with *International Classification of Diseases, Ninth *and* Tenth Revisions, Clinical Modification* (ICD-9-CM and ICD-10-CM) codes, which allows more specific categorization of defects than that permitted by ICD-CM codes alone. To increase completeness of case ascertainment, the Registry conducts hospital audits and links to the Statewide Planning and Research Cooperative System (SPARCS) administrative discharge database. SPARCS is an administrative all-payer data-reporting system that collects information on hospital discharges, services, and treatments using ICD-CM codes. SPARCS requires monthly submission of billing data, which is beneficial for timely prospective surveillance; however, it does not collect narrative descriptions of birth defects (*8*,*9*). The contribution of hospital requests compared with querying administrative discharge databases to ascertain cases of microcephaly is unknown, but might inform prospective surveillance methods.

Although congenital microcephaly is a reportable birth defect and should be included in hospital reports to the Registry, because of concerns about timeliness and completeness of routine reporting, a query was sent to all 154 New York birth hospitals. Hospitals were asked to report all newborns who had a diagnosis code specifying microcephaly (ICD-9-CM code 742.1 or ICD-10-CM code Q02), born during 2013–2015 to women who resided in New York at the time of delivery. All birth hospitals responded, and 83 (54%) identified suspected cases of microcephaly. The SPARCS database was also queried for ICD-9-CM code 742.1 or ICD-10-CM code Q02. Charts were obtained for all newborns with suspected microcephaly and were reviewed by trained clinicians who abstracted birth measurements (head circumference, length, and weight), demographic information, and prespecified infant clinical characteristics, maternal conditions, and maternal/fetal exposures that might have associations with birth defects. Maternal conditions included preeclampsia, eclampsia, hypertension, and gestational diabetes. Prenatal exposures included in utero and perinatal infections (including infections with *Toxoplasma gondii*, rubella virus, cytomegalovirus, herpes simplex virus, human immunodeficiency virus, *Treponema pallidum*, and varicella, dengue, and lymphocytic choriomeningitis viruses), maternal consumption of alcohol, tobacco, illicit drugs and certain teratogenic medications (warfarin, retinoic acid, anticonvulsants, and angiotensin-converting enzyme [ACE] inhibitors), and environmental exposures (i.e., radiation, lead, and mercury). Information also was abstracted on any documented genetic anomalies such as trisomy, gene deletions or duplications, and genomic imprinting, and on family history of birth defects, and parental consanguinity.

Suspected congenital microcephaly was classified according to CDC/NBDPN case definitions (*2*). Overall microcephaly included all physician diagnoses of microcephaly, regardless of the head circumference percentile. Cases of severe congenital microcephaly were defined as the INTERGROWTH-21st^§^ head circumference <3rd percentile for gestational age and sex. Statewide total and severe congenital microcephaly prevalence, and prevalence by health service area^¶^ were calculated using the number of live births during 2013–2015 as the denominator. Maternal race/ethnicity data were obtained by matching with the New York State Department of Health and New York City Department of Health and Mental Hygiene vital records databases, and supplemented with race and ethnicity from SPARCS when data were missing from vital records.

A total of 529 suspected cases of microcephaly were identified from the two sources (Table 1). Of the total 529 suspected cases, 499 (94%) met the overall microcephaly case definition. Thirty (6%) did not meet a case definition because of misclassification (e.g., macrocephaly or microphallus), or because both a physician diagnosis and anthropometric information necessary to accurately categorize head circumference percentile were missing. Among the 499 newborns meeting the overall microcephaly case definition, the majority were identified by both sources; 470 (94%) were identified by hospital requests and 454 (91%) by SPARCS query. A subset of 284 (54%) newborns met the case definition for severe congenital microcephaly, 263 (93%) of whom were identified by hospital requests and 254 (90%) by SPARCS query.

**TABLE 1:** Microcephaly case counts by source of information and National Birth Defects Prevention Network (NBDPN) case definition — New York, 2013–2015

Source of information	No. of suspected microcephaly cases	Confirmed* cases classified by NBDPN case definition	
		Overall microcephaly^†^	Severe congenital microcephaly^§^
		No. (%)	No. (%)
Hospital request or SPARCS database	529	499 (100)	284 (100)
Hospital request	495	470 (94)	263 (93)
SPARCS database	472	454 (91)	256 (90)

**Abbreviation:** SPARCS = Statewide Planning and Research Cooperative System.

* Confirmed by retrospective chart review.

^†^ NBDPN case definition for overall microcephaly: all physician diagnoses of microcephaly, regardless of head circumference percentile.

^§^ NBDPN case definition for severe congenital microcephaly: head circumference <3rd percentile for gestational age and sex.

During 2013–2015, the overall prevalence of microcephaly in New York was 7.4 per 10,000 live births, and the prevalence of severe congenital microcephaly was 4.2 per 10,000 live births, with elevated prevalence of severe congenital microcephaly noted in Western New York (7.2) and Finger Lakes (5.9) health service areas (Table 2). The majority of newborns with severe congenital microcephaly were in New York City (162, 57%), and the prevalence in New York City (4.8 per 10,000 live births) was similar to the statewide prevalence.

**TABLE 2:** Prevalence of severe congenital microcephaly,*^,†^ by health service area — New York, 2013–2015

Health service area	No. of patients with microcephaly^§^	No. of births^¶^	No. of cases per 10,000 live births
**All areas**	**284**	**673,077**	**4.2**
Western New York	33	45,914	7.2
Finger Lakes	23	39,301	5.9
Central New York	16	45,412	3.5
NY-Penn	2	8,547	2.3
Northeastern New York	7	40,676	1.7
Mid-Hudson	22	70,512	3.1
New York City	162	336,047	4.8
Nassau-Suffolk	19	86,668	2.2

* Confirmed by retrospective chart review and classified by National Birth Defects Prevention Network (NBDPN) case definition.

^†^ NBDPN case definition for severe congenital microcephaly: head circumference <3rd percentile for gestational age and sex.

^§^ Cases ascertained from 1) responses to a query of all 154 New York birth hospitals and 2) query of Statewide Planning and Research Cooperative System administrative discharge database for all newborns with diagnosis code specifying microcephaly (*International Classification of Diseases, Ninth Revision, Clinical Modification* code 742.1 or *International Classification of Diseases, Tenth Revision, Clinical Modification* code Q02), born during 2013–2015 to women who resided in New York at the time of delivery.

^¶^ Number of live births obtained from the New York State Department of Health Vital Records.

Approximately equal proportions of newborns with severe congenital microcephaly were Hispanic (31%), non-Hispanic white (30%), and non-Hispanic black (30%) (Table 3). The majority (165 of 284, 58%) of mothers of newborns with severe congenital microcephaly had a pregnancy risk factor or a birth risk factor, including 57 (20%) with a pregnancy complication, 46 (16%) with an in utero or perinatal infection, and 54 (19%) who consumed alcohol, tobacco, illicit drugs, or teratogenic medications during pregnancy. Smaller numbers of infants had a confirmed genetic anomaly (37, 13%), family history of birth defects (20, 7%), or were the result of parental consanguinity (7, 2%).

**TABLE 3:** Selected characteristics for cases (N = 284) of severe congenital microcephaly*^,†^ — New York, 2013–2015

Characteristic	No. (%)
**Infant**	
**Sex**	
Female	163 (57)
Male	121 (43)
**Gestational age**	
Term (≥37 wks)	193 (68)
Preterm (<37 wks)	88 (31)
Missing	3 (1)
**Birth weight**	
Normal weight (>2,500 g)	116 (41)
Low birth weight (1,500–2,500 g)	138 (49)
Very low birth weight (<1,500 g)	30 (11)
**Plurality**	
Singleton	264 (93)
Twin	18 (6)
Triplet or more	2 (1)
**Maternal**	
**Age group (yrs)**	
<35	221 (78)
≥35	56 (20)
Missing	7 (2)
**Race/Ethnicity^§^**	
Hispanic	87 (31)
Black, non-Hispanic	84 (30)
White, non-Hispanic	84 (30)
Asian, non-Hispanic/Other/Missing	29 (10)
**Received prenatal care**	
Yes	241 (85)
No	7 (2)
Missing	36 (13)
**Complications, exposures, genetic disorders, and family history^¶^**	
None	119 (42)
Any	165 (58)
Pregnancy complications**	
Any	57 (20)
Preeclampsia	22 (8)
Gestational diabetes	12 (4)
**In utero or perinatal infections**	
Any infection^††^	46 (16)
Maternal herpes simplex virus	9 (3)
Infant cytomegalovirus infection	10 (4)
**Teratogenic exposures**	
Any	54 (19)
Alcohol	7 (2)
Tobacco	30 (11)
Illicit drugs	33 (12)
Teratogenic medications^§§^	3 (2)
**Environmental exposure**	
Any	1 (<1)
Radiation	0 (—)
Lead	1 (<1)
Mercury	0 (—)
**Confirmed genetic anomaly in newborn^¶¶^**	37 (13)
**Family history of birth defects**	20 (7)
**Parental consanguinity**	7 (2)

* Confirmed by retrospective chart review and classified by National Birth Defects Prevention Network (NBDPN) case definitions.

^†^ NBDPN case definition for severe congenital microcephaly: head circumference <3rd percentile for gestational age and sex.

^§^ Maternal race and ethnicity variables primarily obtained from Vital Records and secondarily from the Statewide Planning and Research Cooperative System database.

^¶^ Not mutually exclusive.

** Including preeclampsia, eclampsia, hypertension, and gestational diabetes.

^††^ Including infections with *Toxoplasma gondii*, rubella virus, cytomegalovirus, herpes simplex virus, human immunodeficiency virus, *Treponema pallidum*, and varicella, dengue, and lymphocytic choriomeningitis viruses.

^§§^ Including warfarin, angiotensin-converting-enzyme (ACE) inhibitors, retinoic acid, and anticonvulsants.

^¶¶^ Documentation of any confirmed genetic anomaly such as trisomy, and gene deletions or duplications, or genomic imprinting.

## Discussion

Before evidence of importation of Zika virus infections, the overall prevalence of microcephaly in New York was 7.4 per 10,000 live births, similar to national estimates for the period 2009–2013 reported recently (*7*), and the prevalence of severe congenital microcephaly was 4.2 per 10,000 live births. The findings in this report highlight the value of confirmation of severe congenital microcephaly using anthropometric measurements to apply the NBDPN case definitions. Use of standardized case definitions allows public health officials to estimate the baseline prevalence of severe congenital microcephaly, a condition that has been observed in infants with congenital Zika virus syndrome, so that comparisons over time and across jurisdictions are possible.

Use of administrative discharge data can enhance case finding for birth defects surveillance, although it is not yet available in many states and, when available, is not always utilized (*10*). Although the vast majority of 284 cases of severe congenital microcephaly were detected by requests to hospital facilities, administrative data identified an additional 7% of cases.

A substantial proportion of newborns with severe congenital microcephaly (42%) identified in this analysis did not have any known maternal conditions or maternal/fetal exposures documented in the newborn hospital record. When the race/ethnicity of mothers of infants with severe congenital microcephaly was compared with the race/ethnicity of live births statewide in New York during 2013–2015, a higher proportion of infants with severe congenital microcephaly were born to Hispanic (31% compared with 23%) and non-Hispanic black mothers (30% compared with 15%) and a lower proportion to non-Hispanic white mothers (30% compared with 48%).** Further investigation is needed to better understand risk factors for microcephaly and disparities in microcephaly prevalence (*7*).

The findings in this report are subject to at least four limitations. First, case finding was limited to live births and did not include stillbirths and terminations, which can account for up to one third of birth outcomes in New York. Second, differences in technique and possible recording errors might have affected the accuracy of the anthropometric measurements documented in the newborn medical record. Third, documentation of various maternal and infant conditions and exposures might be incomplete in the newborn medical record and could result in underascertainment of these characteristics. Finally, although the possibility of Zika-associated microcephaly prior to 2016 cannot be excluded, it is unlikely to have contributed substantially to the prevalence of congenital microcephaly in New York during 2013–2015.

Collaboration between state and local health departments was essential for rapidly obtaining and validating medical records. In addition, increased collaboration and coordination between public health professionals and health care providers in improving processes for head circumference measurement and documentation of diagnoses can help improve accuracy of future estimates of microcephaly prevalence. Clinical documentation of maternal travel histories in the charts of newborns with birth defects will allow for retrospective identification of possible Zika virus exposure in utero. The 2013–2015 New York prevalence estimate of severe congenital microcephaly will enable comparison with future severe congenital microcephaly prevalence estimates and estimation of attributable risk after Zika virus importation.

**Summary**

**What is already known about this topic?**

Zika virus infection during pregnancy can cause severe congenital microcephaly. In New York, the baseline prevalence of severe congenital microcephaly (defined by CDC and the National Birth Defects Prevention Network as head circumference <3rd percentile for gestational age and sex) has not been known.

**What is added by this report?**

During 2013–2015, before documentation of widespread introduction of imported Zika virus infection in the continental United States, the prevalence of severe congenital microcephaly in New York was 4.2 per 10,000 live births. Requests to birth hospitals identified 93% of cases, and statewide administrative discharge data identified 90% of cases.

**What are the implications for public health practice?**

Administrative data can enhance microcephaly case finding for birth defects surveillance programs. Cases of congenital microcephaly must be clinically confirmed using anthropometric measurements to determine whether they meet the case definition for severe congenital microcephaly. A baseline prevalence estimate of severe congenital microcephaly can enable estimation of risk attributable to Zika virus infection.

## Footnotes

* These authors contributed equally to this report.

† Travelers returning from affected areas, their sexual contacts, or infants infected in utero.

§ http://intergrowth21.ndog.ox.ac.uk/.

¶ A health service area is a single county or a cluster of counties that are relatively self-contained with respect to hospital care.
